# Comparison of Conventional versus Steerable-Catheter Guided Coronary Sinus Lead Positioning in Patients Undergoing Cardiac Resynchronization Device Implantation

**DOI:** 10.1371/journal.pone.0143292

**Published:** 2015-11-23

**Authors:** Fikret Er, Dilek Yüksel, Martin Hellmich, Natig Gassanov

**Affiliations:** 1 Department of Internal Medicine I, Klinikum Gütersloh, University Hospital Münster, Gütersloh, Germany; 2 Institute of Medical Statistics, Informatics and Epidemiology, University of Cologne, Cologne, Germany; University of Messina, ITALY

## Abstract

**Objectives:**

The aim of this study was to compare conventional versus steerable catheter guided coronary sinus (CS) cannulation in patients with advanced heart failure undergoing cardiac resynchronization therapy (CRT).

**Background:**

Steerable catheter guided coronary sinus cannulation could reduce fluoroscopy time and contrast medium use during CRT implantation.

**Methods:**

176 consecutive patients with ischemic and non-ischemic heart failure undergoing CRT implantation from January 2008 to December 2012 at the University Hospital of Cologne were identified. During the study period two concurrent CS cannulation techniques were used: standard CS cannulation technique (standard-group, n = 113) and CS cannulation using a steerable electrophysiology (EP) catheter (EPCath-group, n = 63). Propensity-score matched pairs of conventional and EP-catheter guided CS cannulation made up the study population (n = 59 pairs). Primary endpoints were total fluoroscopy time and contrast medium amount used during procedure.

**Results:**

The total fluoroscopy time was 30.9 min (interquartile range (IQR), 19.9–44.0 min) in the standard-group and 23.4 min (IQR, 14.2-34-2 min) in the EPCath-group (p = 0.011). More contrast medium was used in the standard-group (60.0 ml, IQR, 30.0–100 ml) compared to 25.0 ml (IQR, 20.0–50.0 ml) in the EPCath-group (P<0.001).

**Conclusions:**

Use of steerable EP catheter was associated with significant reduction of fluoroscopy time and contrast medium use in patients undergoing CRT implantation.

## Introduction

Cardiac resynchronization therapy (CRT) is recommended in patients with heart failure and QRS delay [1, 2]. The beneficial effects on morbidity and mortality of CRT have been clearly demonstrated in several large randomized trials [3–5].

CRT device implantation has been broadly established [6]. Most of the technical implantation procedure is similar to conventional pacemaker and defibrillator implantation. Atrial and right ventricular leads and defibrillator coils are implanted in common transvenous pacemaker implantation techniques [7]. The challenging part during CRT device implantation is the coronary sinus (CS) cannulation and lead positioning for left ventricular pacing. These steps mainly trigger fluoroscopy time, contrast medium use and total procedure duration. Alternative CS cannulation and lead implantation could increase the efficiency, reduce radiation and contrast agent use.

## Methods

### Inclusion criteria

Patients with transvenous implantation of de novo cardiac resynchronization therapy (CRT) with or without defibrillator (CRT-D vs. CRT-P) from January 2008 to December 2012 at the University Hospital of Cologne were identified. All patients had guideline-conform indication for CRT. Data of experienced physicians, defined as previous implantation of 20 or more CRT devices, were included. Physician used the EP catheter were confident with EP catheters and skilled in EP procedures.

### Retrograde coronary sinus cannulation

**Fig 1:**
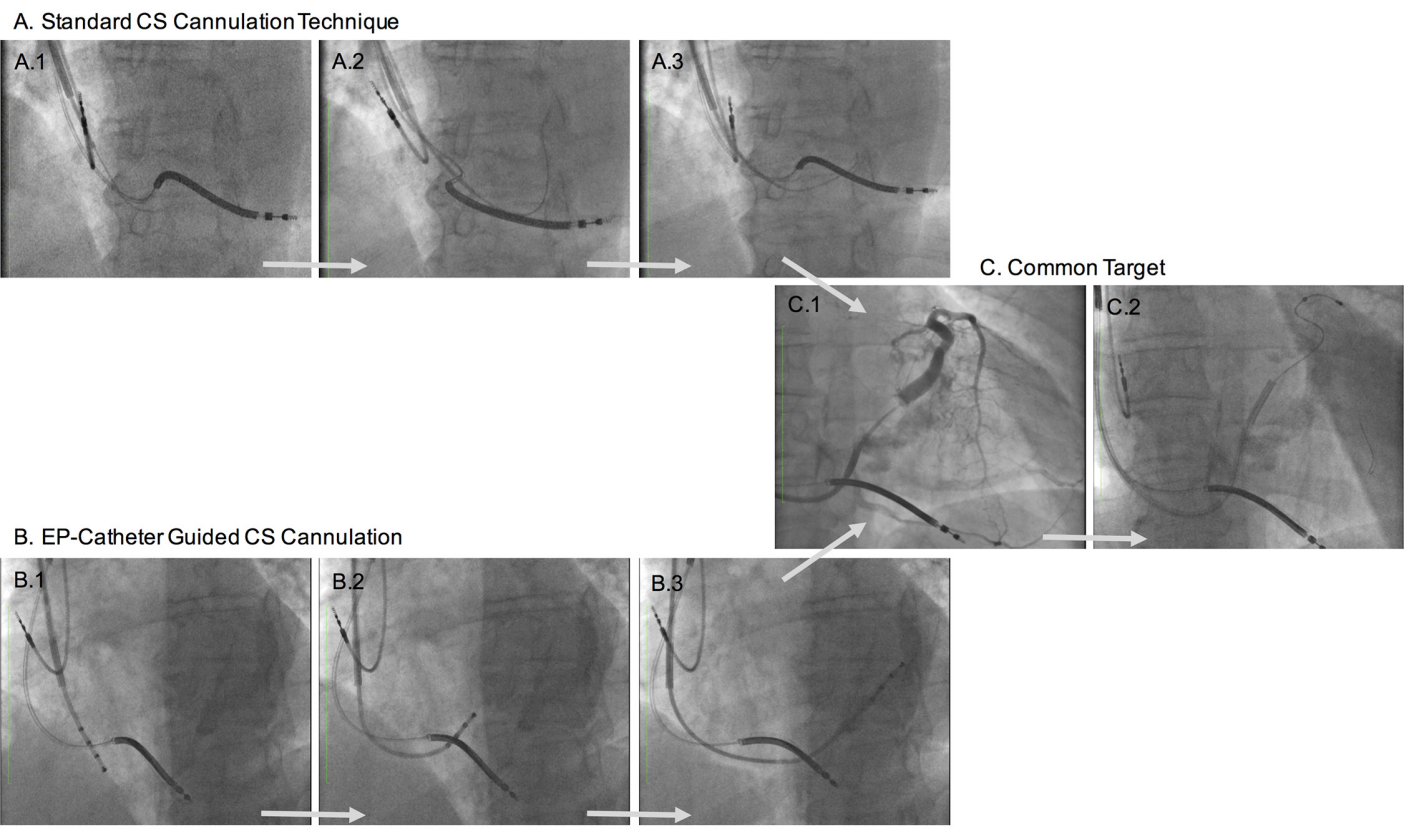
Original X-ray showing CS cannulation in LAO 30° view. 1. **A.1-3.** Standard cannulation technique using preformed sheets and hydrophilic wire. **B.1-3.** Steerable EP catheter with exact cannulation. **C.1-2.** CS angiogram and coronary wire guided LV-lead positioning.

Conventional coronary sinus cannulation was performed using standard, not selectively steerable preformed sheets, multi purpose or other coronary catheter and different coated wires. This method was compared with retrograde CS cannulation using full steerable EP catheter (Polaris, Boston Scientific) electrophysiology catheter and standard sheet (Attain command catheter, Medtronic; Fig 1). Intracardial electrographic signals were not used for localization and access the CS.

### Data collection and study endpoints

All patients receiving CRT during study period were identified and the electronic patient charts and implantation records were retrospectively analyzed. During implantation procedure total fluoroscopy time was electronically calculated and digitally recorded. Contrast medium amount was manually calculated and digitally recorded. All other procedure relevant data were covered by implantation sheet.

### Ethics

Patients data was anonymized, analyzed retrospectively and de-identified prior to analysis. In accordance to local law retrospective analysis requires no ethics votum and no written informed consent. ClinicalTrials.gov Identifier NCT01922544.

### Statistical methods

Qualitative variables were summarized by count (percentage), quantitative variables by mean (standard deviation) or median (interquartile range), contingent on distributional characteristics (e.g. skewness). A propensity score, i.e. the probability of having received EPCath, was derived by multiple logistic regression including the variables age, body-mass-index, ischemic cardiomyopathy, myocardial infarction number of implanted leads and use of defibrillator. Patients from both treatment groups were then matched on the propensity score with tolerance ±0.05. Groups were compared either by means of exact Fisher, two-sample *t*-, and exact Wilcoxon rank sum test (n = 113 vs. n = 63) or exact binomial, paired t-, and exact Wilcoxon signed rank test (n = 59 pairs), respectively. Calculations were done with SPSS Statistics software (IBM Corp., Armonk, NY, USA).

## Results

**Fig 2:**
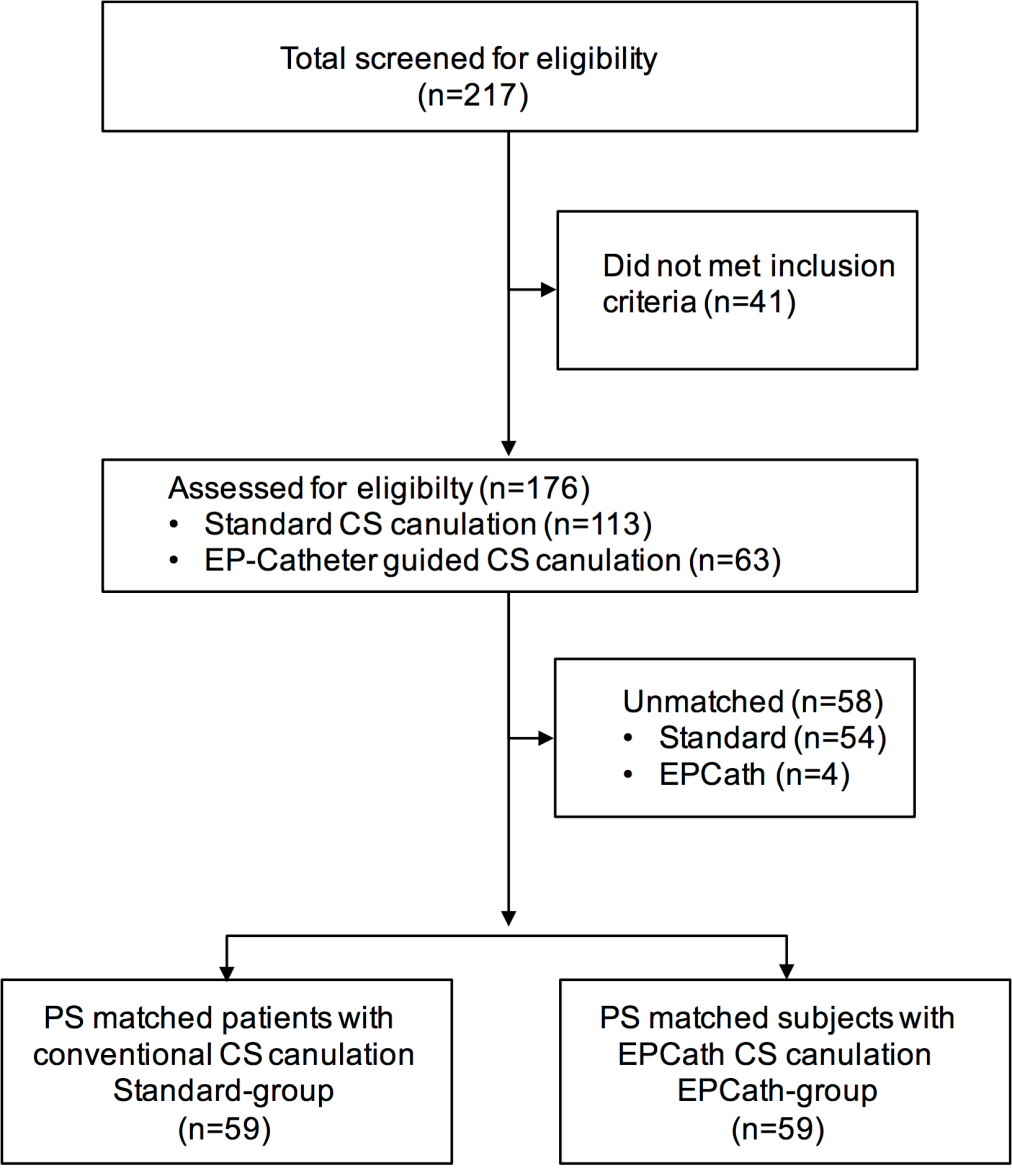
Study flow chart.

During study period 217 subjects with CRT device implantation were identified. Of these 25 subjects were excluded because of previous device implantation and 16 due to less experienced physician. 176 subjects were included into analysis. Using propensity score matching data of 59 subjects with conventional implantation (standard-group) and 59 subjects undergoing CS cannulation using a steerable EP catheter (EPCath-group) were identified for comparison (Fig 2).

Demographic data of subjects pre- and post-matching are shown in Table 1. In most subjects right atrial, right ventricular and coronary sinus leads were implanted (74.6% in the Standard-group vs. 71.2% in the EPCath-group; p = 0.82).

**Table 1:** Demographic data in standard vs. EPCath-group before and after propensity score matching.

	Before PS matching			After PS matching		
	Standard-group n = 113	EPCath-group n = 63	p	Standard-group n = 59	EPCath-group n = 59	p
Men (%)	33 (29.2)	14 (22.2)	0.38^a^	15 (25.4)	14 (23.7)	1.00^d^
Age (years, mean ± SD)	68.8 ± 9.4	68.7 ± 9.6	0.96^b^	69.0 ± 10.7	68.9 ± 9.8	0.94^e^
BMI, kg/m2, median (IQR)	25.4 (23.4 to 27.9)	24.8 (23.4 to 27.1)	0.35^c^	25.7 (23.5 to 27.7)	24.9 (23.4 to 27.3)	0.93^f^
Ischemic cardiomyopathy (%)	58 (51.3)	33 (52.4)	1.00^a^	41 (69.5)	33 (55.9)	0.13^d^
Myocardial infarction (%)	38 (33.6)	25 (39.7)	0.51^a^	33 (55.9)	25 (42.4)	0.17^d^
Hypertension (%)	73 (64.6)	38 (60.3)	0.63^a^	38 (64.4)	36 (61.0)	0.86^d^
Diabetes mellitus (%)	37 (32.7)	21 (33.3)	1.00^a^	22 (37.3)	21 (35.6)	1.00^d^
3-Lead implantation (%)	81 (71.7)	43 (68.3)	0.73^a^	44 (74.6)	42 (71.2)	0.82^d^
CRT-D (%)	72 (64.9)	55 (87.3)	0.001^a^	53 (89.8)	51 (86.4)	0.50^d^

SD ‘standard deviation’, IQR ‘interquartile range’

^a^ Exact Fisher test

^b^ Two-sample t-test

^c^ Exact Wilcoxon rank sum test

^d^ Exact binomial test

^e^ Paired t-test

^f^ Exact Wilcoxon signed rank test

Total fluoroscopy time was significantly lower in the EPCath-group compared to standard-group (23.4 min (IQR, 14.2 to 34.2) vs. 30.9 min (IQR, 19.0 to 44.0 min) p = 0.011; Table 2). Less contrast agent was used during implantation in the EPCath-group (25.0 ml (IQR 20.0 to 50.0 ml) compared to conventional-group (60.0 ml (IQR 30.0 to 100.0 ml; p<0.001; Table 2). Overall success rate of complete CRT implantation was 93.2% (EPCath-group) vs. 89.8% (standard-group; p = 0.75). In four subjects in the EPCath-group and in four subjects in the standard-group CS cannulation was obtained but no adequate LV-lead position could be achieved. In two subjects in the control-group CS access failed. Major complication like coronary sinus dissection, pericardial effusion or tamponade did not occur in any subjects.

**Table 2:** Primary and secondary endpoints in standard vs. EPCath-group before and after propensity score matching.

	Before PS matching			After PS matching		
	Standard-group n = 113	EPCath-group n = 63	p	Standard-group n = 59	EPCath-group n = 59	p
**Primary endpoints**						
Total fluoroscopy time (min), median (IQR)	29.8 (19.2 to 48.6)	23.4 (14.3 to 33.2)	0.005^a^	30.9 (19.0 to 44.0)	23.4 (14.2 to 34.2)	0.011^c^
Contrast agent amount (ml), median (IQR)	60 (30 to 100)	25 (20 to 50)	<0.001^a^	60 (30 to 100)	25 (20 to 50)	<0.001^c^
**Secondary endpoint**						
Success (%)	103 (91.2)	59 (93.7)	0.77^b^	53 (89.8)	55 (93.2)	0.75^d^

IQR ‘interquartile range’

^a^ Exact Wilcoxon rank sum test

^b^ Exact Fisher test

^c^ Exact Wilcoxon signed rank test

^d^ Exact binomial test

## Discussion

Gaining access to the CS and lead positioning for left ventricular pacing are the most challenging and time-consuming steps during CRT implantation. Indeed, inability to access coronary venous system is the most common cause of implant failure [8]. Therefore, new CS cannulation strategies are required to increase the resynchronization efficiency and to diminish the duration and risks of the operation. Three-dimensional electroanatomic mapping or sensor based electromagnetic tracking systems might be beneficial for CS lead implantation [9, 10]. Both techniques are limited in usability to due the higher costs and availability in catheter laboratories without electrophysiology.

The current study provides the first systematical assessment of steerable catheter cannulation for CS lead implantation. Our single center comparison of two CS probing techniques demonstrates fast and effective CRT implantation using a steerable electrophysiology catheter. The technique of CS accessing using steerable catheter is not novel and has been routinely used during electrophysiological procedures for many years.

In the present study total radiation time was decreased by 24% using steerable EP catheter. As frequency of cardiac procedures using X-ray radiation are rapidly increasing, limitation of total radiation time beside other prevention strategies is essential for both, health care employees and patients. The present study confirms the results demonstrated by Wang et al. where steerable EP catheter and additional use of intracardial signals has been shown to shorten the procedure time [11]. Another major issue in cardiovascular interventions is the necessity of nephrotoxic contrast agent use. Contrast induced nephropathy (CIN) significantly affects morbidity and mortality in patients with impaired renal function [12]. Especially in heart failure patients reduced ejection fraction has been associated with an increased risk for CIN [13, 14]. In this respect, steerable EP catheter may decrease the rate of contrast-induced complications in patients undergoing CRT implantation.

In conclusion, using a new steerable EP catheter significantly decreases procedure and fluoroscopy times, and may become the new standard during CRT implantation. The retrospective character of the study might limit the conclusions, although avoiding bias in technique comparisons is generally challenging even in prospective randomized trials.

## References

[1] Moss AJ, Hall WJ, Cannom DS, Klein H, Brown MW, Daubert JP, et al Cardiac-resynchronization therapy for the prevention of heart-failure events. The New England journal of medicine. 2009;361(14):1329–38. 10.1056/NEJMoa0906431 . 19723701

[2] McMurray JJ, Adamopoulos S, Anker SD, Auricchio A, Bohm M, Dickstein K, et al ESC Guidelines for the diagnosis and treatment of acute and chronic heart failure 2012: The Task Force for the Diagnosis and Treatment of Acute and Chronic Heart Failure 2012 of the European Society of Cardiology. Developed in collaboration with the Heart Failure Association (HFA) of the ESC. European heart journal. 2012;33(14):1787–847. 10.1093/eurheartj/ehs104 . 22611136

[3] Cleland JG, Daubert JC, Erdmann E, Freemantle N, Gras D, Kappenberger L, et al The effect of cardiac resynchronization on morbidity and mortality in heart failure. The New England journal of medicine. 2005;352(15):1539–49. 10.1056/NEJMoa050496 . 15753115

[4] Tang AS, Wells GA, Talajic M, Arnold MO, Sheldon R, Connolly S, et al Cardiac-resynchronization therapy for mild-to-moderate heart failure. The New England journal of medicine. 2010;363(25):2385–95. 10.1056/NEJMoa1009540 . 21073365

[5] Bristow MR, Saxon LA, Boehmer J, Krueger S, Kass DA, De Marco T, et al Cardiac-resynchronization therapy with or without an implantable defibrillator in advanced chronic heart failure. The New England journal of medicine. 2004;350(21):2140–50. 10.1056/NEJMoa032423 . 15152059

[6] Gregoratos G. Indications and recommendations for pacemaker therapy. American family physician. 2005;71(8):1563–70. . 15864898

[7] Kennergren C. Impact of implant techniques on complications with current implantable cardioverter-defibrillator systems. The American journal of cardiology. 1996;78(5A):15–20. . 8820831 10.1016/s0002-9149(96)00497-3

[8] Bax JJ, Abraham T, Barold SS, Breithardt OA, Fung JW, Garrigue S, et al Cardiac resynchronization therapy: Part 2—issues during and after device implantation and unresolved questions. J Am Coll Cardiol. 2005;46(12):2168–82. 10.1016/j.jacc.2005.09.020 . 16360043

[9] Niazi I, Ryu K, Hood R, Choudhuri I, Akhtar M. Three-dimensional electroanatomic mapping of the coronary veins during cardiac resynchronization therapy implant: feasibility and possible applications. Journal of interventional cardiac electrophysiology: an international journal of arrhythmias and pacing. 2014;41(2):147–53. 10.1007/s10840-014-9932-9 . 25005455

[10] Thibault B, Andrade JG, Dubuc M, Talajic M, Guerra PG, Dyrda K, et al Reducing Radiation Exposure during CRT Implant Procedures: Early Experience with a Sensor-Based Navigation System. Pacing and clinical electrophysiology: PACE. 2015;38(1):63–70. 10.1111/pace.12522 . 25311868

[11] Wang L, Yuan S, Borgquist R, Hoijer CJ, Brandt J. Coronary sinus cannulation with a steerable catheter during biventricular device implantation. Scandinavian cardiovascular journal: SCJ. 2014;48(1):41–6. 10.3109/14017431.2013.875623 . 24432887

[12] Er F, Nia AM, Dopp H, Hellmich M, Dahlem KM, Caglayan E, et al Ischemic preconditioning for prevention of contrast medium-induced nephropathy: randomized pilot RenPro Trial (Renal Protection Trial). Circulation. 2012;126(3):296–303. 10.1161/CIRCULATIONAHA.112.096370 . 22735306

[13] Ando G, Morabito G, de Gregorio C, Trio O, Saporito F, Oreto G. Age, glomerular filtration rate, ejection fraction, and the AGEF score predict contrast-induced nephropathy in patients with acute myocardial infarction undergoing primary percutaneous coronary intervention. Catheterization and cardiovascular interventions: official journal of the Society for Cardiac Angiography & Interventions. 2013;82(6):878–85. 10.1002/ccd.25023 . 23703775

[14] Capodanno D, Ministeri M, Dipasqua F, Dalessandro V, Cumbo S, Gargiulo G, et al Risk prediction of contrast-induced nephropathy by ACEF score in patients undergoing coronary catheterization. Journal of cardiovascular medicine. 2014 10.2459/JCM.0000000000000215 . 25304032

